# Supplementary material for: Temporal Alignment of Dual Monitor Accelerometry Recordings
Source: Sensors (Basel). 2021 Jul 13;21(14):4777. doi: 10.3390/s21144777 (PMC8309758; doi:10.3390/s21144777)
Supplement: Supplementary file 1 [file sensors-21-04777-s001.zip › sensors-1272594-supplementary.pdf]

Table S1. Reference values, estimated and absolute differences in seconds (number of samples) for the initial offset, and day seven accumulated drift with the subjects in the WH group.

Reference		Estimated		Absolute Difference	
Offset	Day 7 drift	Offset	Day 7 Drift	Offset	Day 7 Drift
−0.73	−8.45	−0.77	−8.39	0.04 (1)	0.06 (2)
−0.32	6.73	−0.43	7.05	0.11 (3)	0.32 (10)
0.74	5.58	0.72	5.59	0.02 (1)	0.01 (0)
3.25	−15.42	3.06	−15.16	0.18 (6)	0.26 (8)
−1.40	17.69	−1.84	17.94	0.43 (13)	0.25 (7)
−6.17	22.13	−6.20	21.77	0.03 (1)	0.36 (11)
−1.84	−1.10	−1.96	−1.08	0.12 (4)	0.02 (1)
0.27	8.24	0.47	8.18	0.19 (6)	0.06 (2)
−2.39	10.05	−1.67	9.69	0.73 (22)	0.36 (11)
0.15	−14.52	−0.02	−14.18	0.16 (5)	0.34 (10)
−1.93	11.15	−1.94	10.81	0.00 (0)	0.33 (10)
−0.94 (2.34)	3.82 (12.38)	−0.96 (2.30)	3.84 (12.23)	0.18 (0.29)	0.22 (0.27)

Table S2. Reference values, estimated and absolute differences in seconds (number of samples) for the initial offset, and day seven accumulated drift with the 30 subjects randomly selected from the TH group.

Reference		Estimated		Absolute Difference	
Offset	Day 7 Drift	Offset	Day 7 Drift	Offset	Day 7 Drift
−0.27	−2.41	−0.27	−2.48	0.00 (0)	0.07 (2)
0.51	2.61	0.38	2.45	0.13 (4)	0.16 (5)
5.62	9.57	5.60	9.29	0.02 (0)	0.28 (8)
4.28	8.05	4.35	7.20	0.07 (2)	0.85 (26)
9.76	17.87	9.89	17.62	0.12 (4)	0.26 (8)
−0.22	−1.35	−0.28	−1.36	0.07 (2)	0.01 (0)
0.65	4.08	0.72	3.84	0.06 (2)	0.25 (7)
−1.04	−2.76	−1.07	−2.68	0.03 (1)	0.08 (3)
−4.11	453.11	−0.90	441.33	3.21 (96)	11.78 (353)
−5.87	−17.43	−5.90	−17.44	0.04 (1)	0.01 (0)
−6.94	−22.22	−7.13	−21.93	0.19 (6)	0.29 (9)
−0.35	−1.55	−0.36	−1.53	0.01 (0)	0.02 (1)
2.64	14.37	2.54	14.35	0.10 (3)	0.02 (1)
1.54	8.16	1.44	8.12	0.09 (3)	0.04 (1)
−0.91	−7.39	−0.92	−7.23	0.00 (0)	0.16 (5)
−2.50	−19.27	−2.62	−19.08	0.12 (4)	0.19 (6)
−3.53	−23.07	−3.58	−22.95	0.06 (2)	0.12 (4)
0.00	0.00	−0.11	0.12	0.11 (3)	0.12 (3)
−5.52	−13.03	−5.73	−12.78	0.21 (6)	0.25 (7)
0.42	1.55	0.40	1.48	0.02 (1)	0.08 (2)
−0.22	−3.82	−0.35	−3.69	0.12 (4)	0.14 (4)
−0.72	−1.49	−0.82	−1.37	0.10 (3)	0.13 (4)
0.55	0.97	0.20	1.27	0.36 (11)	0.30 (9)
0.12	4.96	−0.01	4.90	0.14 (4)	0.06 (2)
1.33	3.90	1.29	3.85	0.04 (1)	0.05 (2)
−3.53	−16.46	−3.95	−15.96	0.42 (12)	0.50 (15)
−2.27	−10.62	−2.55	−10.33	0.28 (9)	0.29 (9)
0.67	0.00	0.59	0.09	0.07 (2)	0.09 (3)
2.05	1.81	1.50	2.96	0.55 (16)	1.15 (35)
−3.92	−7.02	−3.98	−6.94	0.06 (2)	0.09 (3)
−0.39 (3.43)	12.70 (83.81)	−0.39 (3.41)	12.37 (81.64)	0.23 (0.62)	0.59 (2.19)
